# Topical Injection of Tranexamic Acid *via* a Drain Plus Drain‐Clamping to Reduce Blood Loss in Degenerative Lumbar Scoliosis Surgery

**DOI:** 10.1111/os.12583

**Published:** 2019-12-18

**Authors:** Jin‐qian Liang, Tian‐hua Rong, Hong‐zhe Liu, Ming‐sheng Tan, Hong Zhao, Xiang‐yang Liu, Lei Chang

**Affiliations:** ^1^ Department of Orthopedics, Peking Union Medical College Hospital, Chinese Academy of Medical Science Peking Union Medical College Beijing China; ^2^ Department of Spine Union Hunan Provincial People's Hospital Hunan China; ^3^ The Orthopaedic Department 2 China‐Japan Friendship Hospital Beijing China

**Keywords:** Blood loss, Degenerative lumbar scoliosis, Drain‐clamping, Topical retrograde injection, Tranexamic acid

## Abstract

**Objective:**

The aim of the present study was to investigate whether an innovative way of administering tranexamic acid (TXA), that is, injecting it retrogradely through the drain and clamping it for 1 h, can reduce postoperative bleeding after degenerative lumbar scoliosis surgery.

**Methods:**

Sixty degenerative lumbar scoliosis patients who underwent posterior lumbar decompression with fusion of three or more levels were retrospectively enrolled and categorized into three groups (TXA, Gelfoam, and control groups). The demographic distribution, operative parameters, length and amount of Hemovac drainage, blood transfusion rate, length of stay, laboratory results (complete blood count and coagulogram), and the postoperative complications were collected and analyzed.

**Results:**

The age of patients in the Gelfoam group was significantly younger than in the TXA and control groups (59.75 ± 6.95 *vs* 66.10 ± 8.80, *P* = 0.016 and 59.75 ± 6.95 *vs* 67.90 ± 5.33, *P* = 0.000, respectively). There were no significant differences in sex, body mass index, comorbid medical status, and operation level between each of the two groups. The three groups did not differ significantly in estimated blood loss during surgery, the mean red blood cell transfusion requirement during hospitalization, and the entire perioperative allogenic blood transfusion rate. The postoperative total blood loss and total drainage were lower in the TXA group than in the control group (1027.14 ± 466.56 *vs* 1390.07 ± 314.85 mL, *P* = 0.006; 322.20 ± 187.32 *vs* 605.50 ± 184.70 mL, *P* = 0.000, respectively). The length of drainage retention in the TXA group was significantly shorter than in the Gelfoam and control groups (46.10 ± 9.00 *vs* 68.00 ± 12.31 h, *P* = 0.000 and 46.10 ± 9.00 *vs* 76.40 ± 10.97 h, *P* = 0.000, respectively). The TXA and Gelfoam groups also had significantly shorter hospital stays than the control group (7.50 ± 0.95 *vs* 9.80 ± 2.44 days, *P* = 0.000, and 7.90 ± 1.16 *vs* 9.80 ± 2.44 days, *P* = 0.003, respectively). At discharge, the mean hemoglobin and hematocrit level were significantly higher in the TXA group compared with the control group (11.77 ± 1.78 g/dL *vs* 10.67 ± 0.94 g/dL, *P* = 0.002; 34.82 ± 3.57% *vs* 31.79 ± 3.85%, *P* = 0.014). No significant difference was identified with respect to prothrombin time, activated partial thromboplastin time, and D‐dimmer among groups (*P* > 0.05). The three groups were comparable in wound problem incidences. Symptomatic deep vein thrombosis and pulmonary embolism were not observed in this study.

**Conclusion:**

Topical injection of TXA retrogradely *via* a drain at the end of a degenerative lumbar scoliosis operation and clamping the drain for an hour can effectively decrease the postoperative blood loss and the length of hospitalization without increasing the complication rate.

## Introduction

Blood loss is unavoidable during and after major spine surgery, especially in degenerative lumbar scoliosis surgery, which usually requires more complicated maneuvers. Allogeneic blood transfusions (ABT) may lead to serious complications, and in recent years, a growing number of effective agents and new techniques for avoiding ABT have been reported^1^. Many methods have been applied to reduce ABT, including autologous blood predonation, antifibrinolytic drugs, acute normovolemic hemodilution, and red blood cell salvage^2^, ^3^. However, the safest and most efficient method remains controversial.

Tranexamic acid (TXA) inhibits tissue fibrinolysis for up to 17 h and, consequently, reduces the possibility of clots entering the extravascular space and accumulating in tissues^4^. It is generally accepted, however, that only a small percentage of intravenously injected TXA reaches the target location. Therefore, a more efficient method (i.e. intra‐articular injection) to deliver TXA is desirable^5^. Another method used to reduce postoperative blood loss is drain‐clamping^6^, ^7^, ^8^. As a strategy for reducing blood loss, drain clamping has been combined with intravenous or intra‐articular administration of TXA and intra‐articular injection of diluted‐epinephrine since approximately 1995, and the preventive effect on blood loss has been well documented^9^, ^10^, ^11^.

We modified that method to make it simpler, easy to use, suitable for major spine surgical patients, and understandable for clinicians. We administered TXA at the end of the operation, injecting it retrogradely through the drain, and then clamped the drain for 1 h to ensure that the TXA stayed at the wound for an adequate amount of time. The efficacy of such topical application of TXA in reducing postoperative bleeding after degenerative lumbar scoliosis surgery has not been reported. Our hypothesis was that topical administration of TXA *via* the drain and then clamping the drain for 1 h would reduce postoperative bleeding after degenerative lumbar scoliosis surgery.

The purpose of the present study was: (i) to test the feasibility of a new way of using TXA (i.e. retrograde injection through the drain followed by 1 h drain clamping); (ii) to evaluate the effectiveness of this method in reducing postoperative blood loss and length of stay; and (iii) to investigate the consequent complications and toxicity of such TXA usage.

## Methods

### *Study Design*

This retrospective clinical trial was conducted at the spine unit of the authors’ hospital between December 2015 and December 2017. It was approved by the Institutional Review Board of our hospital. All patients provided written informed consent for the study and surgery. A total of 60 patients were enrolled and categorized into three groups. The researchers who enrolled the participants and analyzed the data did not take part in patient care and assessment. Research data were collected on demographics, preoperative investigations, blood loss, and blood products transfused during surgery.

### *Inclusion and Exclusion*

The inclusion criteria were as follows:

(i) Definite diagnosis of degenerative lumbar scoliosis.
(ii) The patient had undergone posterior lumbar decompression and fusion of three or more levels.
(iii) The patients were willing to participate after giving written informed consent.
(iv) Complete clinical data of the patient should be obtainable.

Exclusion criteria were defined as follows:

(i) Allergy to TXA.
(ii) All‐cause anemia before the operation (male hemoglobin <13 g/dL, female hemoglobin <12 g/dL).
(iii) Any type of coagulopathy.
(iv) Being medicated with anticoagulants and/or antiplatelet agents.
(v) Previous history of any thromboembolic events (deep vein thrombosis, ischemic heart disease, pulmonary embolism, transient ischemic attack, stroke, or subarachnoid hemorrhage).
(vi) Renal insufficiency (creatinine >2.0 mg/dL), chronic liver disease, and pregnancy.

### *Intervention*

All patients received standard general anesthesia. Minute ventilation was set to maintain normocapnia. A standard posterior subperiosteal exposure of the spine, decompression, and instrumentation with bone grafting were performed with a similar operative technique among this patients. Surgery was performed by two senior surgeons. Controlled hypotension was applied for all patients in the groups with mean arterial blood pressure (MAP) of approximately 20% below the preoperative value, with a minimum MAP of 60 mmHg. Controlled hypotension was maintained until nearly completing the procedure, and then MAP was returned to the baseline pressure to check for bleeding. Meticulous hemostasis was achieved at the end of the procedure with use of bipolar electrocautery.

In the two experimental groups (TXA and Gelfoam groups), after adequate hemostasis had been achieved, retrograde injection of TXA *via* the drain at the end of the operation followed by pushing TXA into the wound by injecting an additional 10 mL of saline and then clamping the drain for 1 h, or a single piece of absorbable gelatin sponge (size, 100 cm^2^) was used, respectively. We designed this clamping interval according to previous data, which indicated that 1 h of clamping was sufficient^12^. In addition, 1 h of clamping is simple for clinicians to apply. The amount of TXA injected was chosen to be one ampoule (10% transamin, 10 mL, 1000 mg; Daiichi‐Sankyo, Tokyo, Japan). This dose was found to be acceptable and effective in previous studies^13^, ^14^. In addition, a single ampoule is a simple quantity and easy for surgeons to administer. In cases in which the wound was small, we cut the piece to such a size that the entire wound could be covered. In cases in which the wound was bigger than the single piece, we cut the piece longitudinally in half and placed the two pieces end to end in the middle of the wound, on top of the bone graft material. We did not place the sponge over the exposed dura, as it can expand in the wound and compress the spinal cord underneath. In both the two experimental groups and the control group, a drain was placed under the deep fascia and the wound was closed in multiple layers. The drained fluid was initially sanguinous and gradually became serous after two to three 8‐h duration.

We followed the principle of transfusion based on the criteria and guidelines for perioperative transfusion suggested by the National Institutes of Health Consensus Conference, which states that the decision to transfuse blood depends on clinical assessment aided by laboratory data indicating that the patient has symptoms and signs associated with acute anemia^15^. Therefore, our indication for blood transfusion was set at a hemoglobin concentration less than 8.5 g/dL or a postoperative hemoglobin level of 8.5–9.0 g/dL with clinical evidence of acute anemia^16^. It was adjusted according to the patient's cardiovascular status.

### *Outcome Measurements*

The primary outcome was perioperative blood loss occurring intraoperatively and 24 h postoperatively. Intraoperative blood loss was defined as the total amount of bleeding before the end of wound closure, which was indirectly calculated from the amount of blood in the suction canisters and that in the soaked lap pads. The blood loss within 24 h after surgery was mainly reflected by the accumulative amount of fluid collected from wound drainage, which was measured and recorded three times a day in 8‐h shifts until drainage tube removal. The drain was routinely removed when the drain output per 8‐h shift was <20 mL.

The secondary outcomes were the estimated total perioperative blood loss and the incidence of autogenic or allogenic blood transfusions. A formula proposed by Nadler *et al*.^17^ and Sehat *et al*.^18^ was used to calculate the total perioperative blood loss. It was based on the maximum postoperative decrease in hemoglobin level adjusted for the weight and height of the patient. Hemoglobin levels were measured on postoperative days (POD) 1 and 3, and day of discharge. The loss of Hb was then estimated according to the following formula:

$$\text{Blood volume}\;\left(L\right)=\text{height}\;{\left(m\right)}^{3}\times 0.356+\text{body weight}\;\left(\mathrm{kg}\right)\times 0.033+0.183\;\left(\text{woman}\right)=\text{height}\;{\left(m\right)}^{3}\times 0.367+\text{body weight}\;\left(\mathrm{kg}\right)\times 0.032+0.604\;\left(\mathrm{man}\right);$$

$${\mathrm{Hb}}_{\text{loss}}\left(g\right)=\text{Blood volume}\times 10\times \left({\mathrm{Hb}}_{i}-{\mathrm{Hb}}_{\mathrm{fin}}\right)+{\mathrm{Hb}}_{t};$$

$$\text{Total blood loss}\;\left(\mathrm{mL}\right)={\mathrm{Hb}}_{\text{loss}}/{\mathrm{Hb}}_{i}\times 100.$$

Hb_loss_ (g) was the amount of Hb lost, Hb_i_ (g/dL) was the Hb concentration before surgery, Hb_fin_ (g/dL) was the Hb concentration on postoperative day 3 or day of discharge (whichever was lower), and Hb_t_ (g) was the total amount of allogeneic and autologous Hb transfused. The transfusion information was retrieved from anesthesia records and postoperative progress notes.

The tertiary outcome was the duration of drainage retention and length of hospital stay. Drainage retention was calculated based on the time of drainage removal minus the time of the end of surgery. Length of hospital stay was calculated by discharge date minus admission date.

Another perspective was coagulation function and related complications. The D‐dimer level was assayed by coagulogram on POD 1 and 3, and day of discharge after surgery. The possibility of deep venous thrombosis (DVT) and/or pulmonary embolism (PE) was observed for 4 weeks after the surgery. The authors also monitored the wound healing condition (e.g. skin necrosis, hematoma, and infection).

### *Statistical Analysis*

The qualitative data were presented as number and percentage. The parametric quantitative data were presented as mean and standard deviation. Comparison between each of the two groups was analyzed by χ^2^‐test or Fisher exact test with Bonferroni adjustment for qualitative data, and the one‐way analysis of variance with *post hoc* test for parametric quantitative data. All statistical analysis was performed using SPSS for Windows Version 22.0 (IBM, NY, USA). *P* < 0.05 was considered statistically significant.

## Results

### *Participants*

A total of 129 patients were eligible for the present study, but 69 patients were excluded because of: simpler procedure (fewer than three levels) 24, anemia 13, they had been medicated with anticoagulants or antiplatelet agents 22, and they had a history of thromboembolic diseases 10. Only 60 patients met the inclusion criteria. Although the patients in the Gelfoam group were significantly younger than in the TXA and control groups (59.75 ± 6.95 *vs* 66.10 ± 8.80, *P* = 0.016 and 59.75 ± 6.95 *vs* 67.90 ± 5.33, *P* = 0.000, respectively), there were no significant differences in sex, BMI, comorbid medical status, and operation level between each of the two groups (Table 1).

**Table 1:** Demographics of the three groups

Characteristics	TXA group (*n* = 20)	Gelfoam group (*n* = 20)	Control group (*n* = 20)
Age (years)	66.10 ± 8.80	59.75 ± 6.95	67.90 ± 5.33
Sex			
Male	7	14	4
Female	13	6	16
BMI (kg/m^2^)	24.87 ± 3.46	26.06 ± 2.74	25.35 ± 3.60
Comorbid medical conditions			
Yes	11	9	9
No	9	11	11
Implanted level	4.10 ± 0.91	4.70 ± 1.13	4.20 ± 0.95

BMI, body mass index; TXA, tranexamic acid.

### *Perioperative Blood Loss*

**Figure 1:**
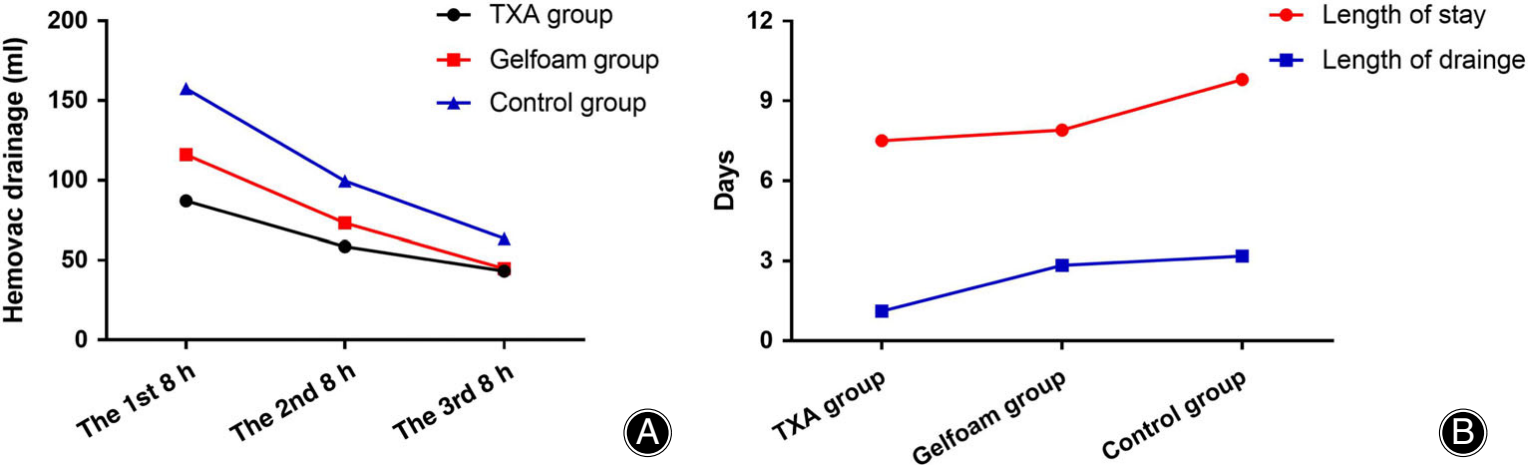
(A) Postoperative Hemovac drainage for each group; and (B) the length of drainage retention and postoperative hospital stay for each group. TXA, tranexamic acid.

As for intraoperative data (Table 2), the three groups did not differ significantly in the estimated blood loss during the operation, the mean red blood cell transfusion requirement during hospitalization, and the entire perioperative allogenic blood transfusion rate. The total blood loss was lower in the TXA group than in the control group (1027.14 ± 466.56 *vs* 1390.07 ± 314.85 mL, *P* = 0.006). The total drainage postoperatively was lower in the TXA group than in the control group (322.20 ± 187.32 *vs* 605.50 ± 184.70 mL, *P* = 0.000). In the TXA, Gelfoam, and control groups, the Hemovac drainage at the first 8‐h point postoperatively was 87.00 ± 41.18, 116.00 ± 39.26, and 157.50 ± 45.52 mL, while Hemovac drainage at the second 8‐h point postoperatively was 58.50 ± 41.07, 73.25 ± 20.28, and 99.50 ± 25.64 mL and at the third 8‐h point postoperatively was 43.25 ± 28.20, 44.75 ± 27.07, and 63.50 ± 23.85 mL, respectively. (Table 2 and Fig. 1).

**Table 2:** Comparing variables in patients with and without the use of topical retrograde tranexamic acid (TXA) injection and drain‐clamping

Characteristics	TXA group (*n* = 20)	Gelfoam group (*n* = 20)	Control group (*n* = 20)
Op time (min)	221.00 ± 22.21	228.50 ± 26.01	240.05 ± 46.39
EBL (mL)	456.00 ± 210.10	567.50 ± 165.65	516.50 ± 241.25
Transfusion (U/pt)	0.75 ± 1.25	0.5 ± 1.28	0.70 ± 0.98
Allogenic blood transfusion, *n* (%)	6 (30.0)	4 (20.0)	7 (35.0)
Hemovac drainage (mL)			
The first 8 h postoperatively	87.00 ± 41.18* ^,^ †	116.00 ± 39.26‡	157.50 ± 45.52
The second 8 h postoperatively	58.50 ± 41.07*	73.25 ± 20.28‡	99.50 ± 25.64
The third 8 h postoperatively	43.25 ± 28.20*	44.75 ± 27.07‡	63.50 ± 23.85
Total drainage	322.20 ± 187.32*	464.00 ± 167.08† ^,^ ‡	605.50 ± 184.70
Total blood loss	1027.14 ± 466.56*	1154.70 ± 609.17	1390.07 ± 314.85(0.006*)
Length of drainage (h)	46.10 ± 9.00* ^,^ †	68.00 ± 12.31‡	76.40 ± 10.97
Hemoglobin (g/dL)			
Baseline	13.17 ± 1.09†	14.75 ± 1.15‡	13.43 ± 0.68
Day 1 postoperation	11.24 ± 1.43	12.25 ± 1.74‡	10.57 ± 1.45
Day 3 postoperation	11.05 ± 1.34*	11.35 ± 1.91‡	10.35 ± 0.62
At discharge	11.77 ± 1.78*	11.07 ± 1.83	10.67 ± 0.94
Hematocrit (%)			
Baseline	39.12 ± 3.21	43.24 ± 3.17	38.76 ± 2.54
Day 1 postoperation	32.57 ± 4.08*	35.12 ± 4.94‡	31.02 ± 4.31
Day 3 postoperation	32.52 ± 3.78*	32.95 ± 5.53‡	29.96 ± 2.31
At discharge	34.82 ± 3.57*	32.13 ± 4.97	31.79 ± 3.85
Postoperative length of stay (d)	7.50 ± 0.95*	7.90 ± 1.16‡	9.80 ± 2.44

EBL, estimated blood loss during operation; Op time, operation time; U blood units, U/pt., units per patient

* *P* < 0.05 between TXA group and Control group

† *P* < 0.05 between TXA group and Gelfoam group

‡ *P* < 0.05 between Gelfoam group and control group.

### *Postoperative Recovery*

**Figure 2:**
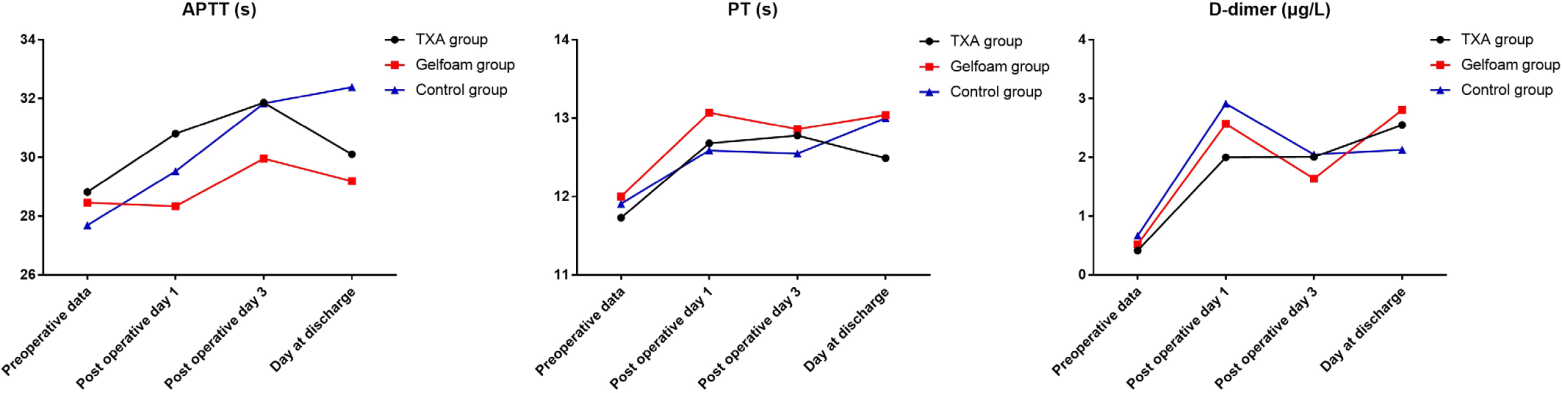
Perioperative coagulation parameters for each group.

The length of drainage retention in the TXA group was significantly shorter than for the Gelfoam and control groups (46.10 ± 9.00 *vs* 68.00 ± 12.31 h, *P* = 0.000 and 46.10 ± 9.00 *vs* 76.40 ± 10.97 h, *P* = 0.000, respectively). The TXA and Gelfoam groups also experienced a shorter length of hospital stay than the control group, with the differences reaching statistical significance (7.50 ± 0.95 *vs* 9.80 ± 2.44 days, *P* = 0.000, and 7.90 ± 1.16 *vs* 9.80 ± 2.44 days, *P* = 0.003, respectively) (Fig. 1). There were no significant differences in the hemoglobin and hematocrit values between patients in TXA and control groups preoperatively. However, at the time of hospital discharge, the mean hemoglobin and hematocrit level were significantly higher in the TXA group compared with the control group (11.77 ± 1.78 g/dL *vs* 10.67 ± 0.94 g/dL, *P* = 0.002; 34.82 ± 3.57% *vs* 31.79 ± 3.85%, *P* = 0.014). As for the perioperative coagulogram, no significant difference was identified with respect to prothrombin time, activated partial thromboplastin time, and D‐dimmer among groups (*P* > 0.05). However, the overall changes of the three parameters showed an upward trend (Fig. 2).

### *Complications*

Two complications were observed in the present study, including one dressing reinforcement in the TXA group and one wound oozing in the control group, both of which occurred within 1 week postoperatively. These two patients were treated by dressing change and had recovered well after 1 week. In all three groups, there were no patients who were readmitted because of postoperative complications within the first 30 days after surgery. There were no infections or clinically identifiable epidural hematomas. Finally, symptomatic deep vein thrombosis and pulmonary embolism were not observed in each group.

## Discussion

The most important finding of the present study was that topical retrograde injection of TXA *via* the drain at the end of the operation followed by clamping the drain for 1 h effectively reduced postoperative blood loss and postoperative hospital stay after major degenerative lumbar scoliosis surgery. This method is simple, easy to perform, suitable for these patients, and understandable for clinicians, despite that the need for blood transfusion was not statistically different between the two groups. The effect of TXA and drain‐clamping in patients undergoing degenerative lumbar scoliosis surgery was certainly clarified, and is similar to that found in previous studies performed in total knee arthroplasty (TKA) 7, 8, 14.

Prolonged postoperative bleeding after posterior spinal procedures may also increase medical expenses or even cause symptomatic epidural hematomas, which may lead to spinal cord compression and subsequent neurologic deficits in severe cases^19^, ^20^, ^21^, ^22^. Furthermore, in patients who were medically and neurologically stable, the primary factor keeping the patient in the hospital was the maintenance of the drain. Liang *et al*.^23^ found that topical use of a TXA‐soaked absorbable gelatin sponge could effectively reduce postoperative blood loss, thus decreasing the length of hospital stay among low‐risk adult patients undergoing lumbar spine surgery. However, the patients in that study were younger and the average surgical levels were much shorter. Ren *et al*.^24^ reported that wound surface soaked with TXA for 5 min before wound closure can significantly reduce postoperative blood loss, accelerate the removal of the drainage tube, and, thus, shorten the duration of hospital stay, without increasing the complication incidence in patients undergoing posterior lumbar spinal fusion surgery. Given that the surgical segments in their study are mostly single‐level, in which the patients have relatively small volume of blood loss, it is underpowered to confirm the assumption that TXA can decrease blood transfusion requirements. In line with the study of Liang *et al*. and Ren *et al*., our study demonstrated that topical retrograde injection of TXA *via* the drain at the end of the operation and then clamping the drain for 1 h could effectively reduce postoperative drain output and the length of hospital stay in multilevel (≥3) posterior lumbar spine surgery for degenerative lumbar scoliosis patients. The possible explanation may be the fact that TXA acts directly to inhibit plasmin activity. Consequently, there is a decrease in proteolytic action on the fibrin monomers and fibrinogen, which results in clot stabilization. This benefit is likely to be of particular importance for elderly patients, and patients those with more operative levels.

Increased perioperative blood loss directly influences the rate of autologous and allogenic blood transfusion, which brings additional risks, including transfusion reactions, infection, and alterations in the coagulation profile of the patients. Application of blood products also has a significant economic impact due to the packaging and storage of autologous blood and the extensive screening process required for allogenic transfusion. Intra‐articular TXA application after TKA has recently been introduced and proved to significantly reduce the postoperative blood loss and allogenic blood transfusion requirement^5^, ^25^. However, in the previous study, we found that the incidence of postoperative blood transfusion was not statistically different between TXA‐soaked absorbable gelatin sponge and control groups^23^. The possible explanations for this negative result may be the relative short operative level and the overall perioperative blood management strategy. Thus, we designed this retrospective clinical trial to investigate the effect of topical TXA application after major spine surgery. The results of the present study also demonstrated no statistically significant differences in the mean red blood cell transfusion requirement during hospitalization, and the entire perioperative allogenic blood transfusion rate between TXA and control groups, suggesting that the main bleeding in the spine surgery occurred in intraoperative episode which was totally different from that in TKA. This finding indicates that meticulous hemostasis during surgery is of great importance in reducing blood loss and allogenic blood transfusion requirement in major spine surgery.

Previous studies have shown the safety and effectiveness of topical application of TXA in spine surgery. Xu *et al*.^26^ reported that no patient developed adverse reactions attributable to the topical administration of TXA and there were no differences in any of the parameters representing wound problems or readmissions within 30 days from discharge between patients with the topical use of TXA and patients in the control group. Similarly, Ren *et al*.^27^ reported that topical use of tranexamic acid can effectively decrease hidden blood loss, without significant complications in adult patients undergoing posterior lumbar spinal fusion surgery. The results of the present study also indicate no statistically significant differences for wound complications between the two cohorts. However, care should be taken before making definite conclusions for these outcomes, as the overall number of wound complications was small.

The present study had some limitations. First, the sample size was small, with only 20 cases being included in each group. As many cases with severe contraindications had been excluded from our trial, it is not comprehensive enough to declare that all the hemostatic measures were safe under all clinical circumstances. In the future, we will perform a further validation on a larger sample size. Second, we did not use TXA intravenously during the operation, which may reduce a large range of intraoperative blood loss. In future, we will perform a further investigation with combined administration of topical and intravenous TXA. Third, investigations using thromboembolism screening tests such as ultrasonography maybe required.

### *Conclusions*

This study provides an important perspective on the management of postoperative bleeding after major spine surgery using topical injection of TXA retrogradely *via* the drain at the end of the operation and clamping the drain for 1 h, which can decrease the postoperative blood loss and length of hospitalization when compared with control models. The topical use of TXA resulted in no toxicity and no serious complications. We believe that our modifications of this technique have made it a simple, easy‐to‐use method that is suitable for use in patients and easy for clinicians to apply.
